# Tsg101 regulates PI(4,5)P_2_/Ca^2+^ signaling for HIV-1 Gag assembly

**DOI:** 10.3389/fmicb.2014.00234

**Published:** 2014-05-20

**Authors:** Lorna S. Ehrlich, Gisselle N. Medina, Sara Photiadis, Paul B. Whittredge, Susan Watanabe, Justin W. Taraska, Carol A. Carter

**Affiliations:** ^1^Molecular Genetics and Microbiology, Stony Brook UniversityStony Brook, NY, USA; ^2^Laboratory of Molecular Biophysics, National Heart Lung and Blood Institute, National Institutes of HealthBethesda, MD, USA

**Keywords:** HIV-1, Gag assembly, L domain, Tsg101, PI(4,5)P_2_, IP3R, Ca^2+^ signaling, ER-PM junction

## Abstract

Our previous studies identified the 1,4,5-inositol trisphosphate receptor (IP3R), a channel mediating release of Ca^2+^ from ER stores, as a cellular factor differentially associated with HIV-1 Gag that might facilitate ESCRT function in virus budding. Channel opening requires activation that is initiated by binding of 1,4,5-triphosphate (IP3), a product of phospholipase C (PLC)-mediated PI(4,5)P_2_ hydrolysis. The store emptying that follows stimulates store refilling which requires intact PI(4,5)P_2_. Raising cytosolic Ca^2+^ promotes viral particle production and our studies indicate that IP3R and the ER Ca^2+^ store are the physiological providers of Ca^2+^ for Gag assembly and release. Here, we show that Gag modulates ER store gating and refilling. Cells expressing Gag exhibited a higher cytosolic Ca^2+^ level originating from the ER store than control cells, suggesting that Gag induced release of store Ca^2+^. This property required the PTAP motif in Gag that recruits Tsg101, an ESCRT-1 component. Consistent with cytosolic Ca^2+^ elevation, Gag accumulation at the plasma membrane was found to require continuous IP3R activation. Like other IP3R channel modulators, Gag was detected in physical proximity to the ER and to endogenous IP3R, as indicated respectively by total internal reflection fluorescence (TIRF) and immunoelectron microscopy (IEM) or indirect immunofluorescence. Reciprocal co-immunoprecipitation suggested that Gag and IP3R proximity is favored when the PTAP motif in Gag is intact. Gag expression was also accompanied by increased PI(4,5)P_2_ accumulation at the plasma membrane, a condition favoring store refilling capacity. Supporting this notion, Gag particle production was impervious to treatment with 2-aminoethoxydiphenyl borate, an inhibitor of a refilling coupling interaction. In contrast, particle production by a Gag mutant lacking the PTAP motif was reduced. We conclude that a functional PTAP L domain, and by inference Tsg101 binding, confers Gag with an ability to modulate both ER store Ca^2+^ release and ER store refilling.

## Introduction

Phosphatidylinositol 4,5-bisphosphate (PI(4,5)P_2_) functions in the cell as plasma membrane anchor and substrate to phospholipase C (PLC) (McLaughlin and Murray, 2005). Together, these two functions give PI(4,5)P_2_ influence over multiple processes in the cell despite being a minor plasma membrane lipid component. Our work and that of others indicate that both functions of PI(4,5)P_2_ are required for HIV-1 virus production, specifically, Gag assembly. Intact PI(4,5)P_2_ at the plasma membrane targets Gag to this site: When PI(4,5)P_2_ is depleted by adventitious expression of 5-ptase IV, the characteristic plasma membrane localization of Gag is not detected (Ono et al., 2004; Chukkapalli et al., 2008; Fernandes et al., 2011); when PI(4,5)P_2_ accumulation is routed to endosomes by adventitious expression of the Arf6/Q67L mutant, Gag is mostly associated with endosomes (Ono et al., 2004). Both conditions are inhibitory to virus particle formation (Ono et al., 2004; Chukkapalli et al., 2008; Fernandes et al., 2011) in agreement with prevailing models that assembly occurs at the plasma membrane (Ehrlich and Carter, 2012; Sundquist and Krausslich, 2012). Underpinning PI(4,5)P_2_-mediated Gag membrane anchoring are direct contacts between PI(4,5)P_2_ and residues in the MA and CA domains in Gag (Saad et al., 2006; Shkriabai et al., 2006). Additionally to this role, our studies (Ehrlich et al., 2010, 2011) demonstrate that Gag assembly requires PI(4,5)P_2_ in its capacity as substrate to PLC. PLC-catalyzed hydrolysis of PI(4,5)P_2_ (Rhee, 2001; Suh et al., 2008) is part of a universal pathway for increasing the concentration of Ca^2+^ ions in the cytosol (Berridge, 2009). We found that inactivation of PLC enzymatic activity abolished localization of Gag to plasma membrane and formation of the assembled Gag particle despite an abundance of intact PI(4,5)P_2_ at the plasma membrane (Ehrlich et al., 2010) while activation of PLC led to enhancement of particle formation (Ehrlich et al., 2011). We interpret this requirement for the function of PI(4,5)P_2_ as PLC substrate to indicate active participation of Ca^2+^ signaling machinery in Gag assembly.

**Figure 1:**
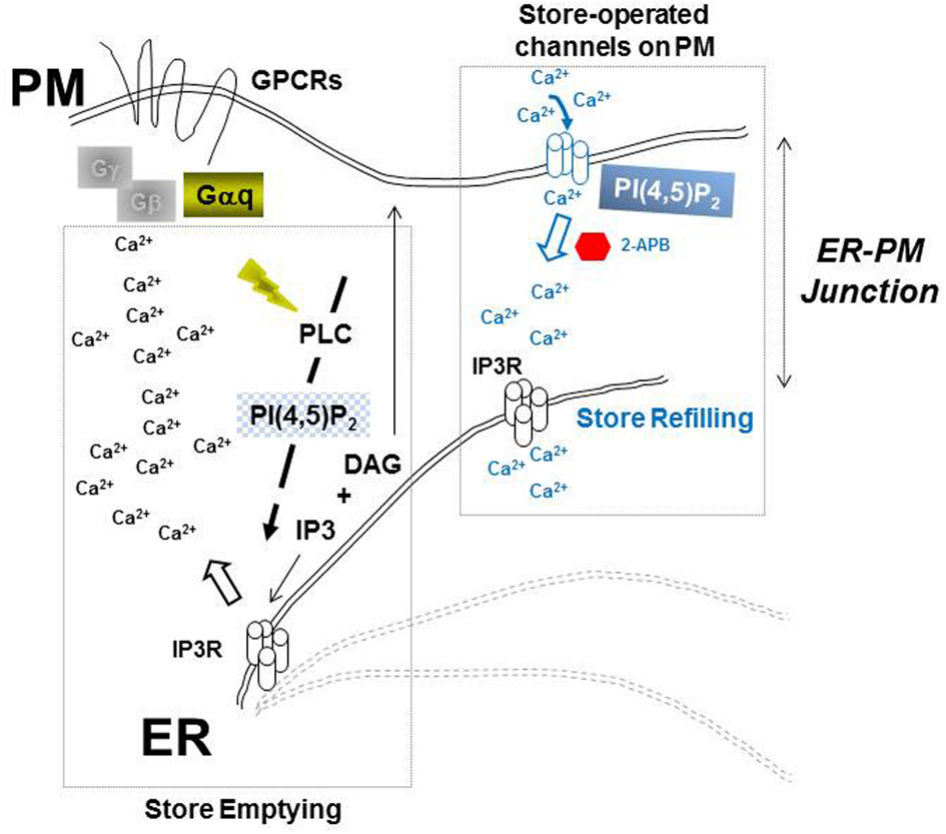
**PI(4,5)P_2_-dependent Ca^2+^ machinery**. Ca^2+^ store emptying (**left**) and store re-filling (**right**) at dynamic ER-PM junctions (dashed lines) are highlighted as key events of IP3R-gating at the prototypical Ca^2+^ store in the ER.

Through a proteomic search aimed at identification of cellular factors that might participate with HIV-1 Gag and endocytic sorting complexes required for transport (*ESCRT*) in facilitating virus budding, we identified inositol (1,4,5)-trisphosphate receptor (IP3R) as a protein enriched with other Ca^2+^ signaling proteins in plasma membrane-enriched sub-cellular fractions when Gag was expressed. Figure 1 outlines the central role of IP3R in PI(4,5)P_2_-dependent Ca^2+^ signaling (Patterson et al., 2004a; Banerjee and Hasan, 2005; Mikoshiba, 2007). IP3R forms a transmembrane Ca^2+^ ion-specific channel on the membrane of the endoplasmic reticulum (ER), the major organelle for intracellular storage of Ca^2+^, where it gates release of the ion into the cytosol. IP3R channel opening requires activation that is initiated by the binding of inositol trisphosphate (IP_3_) which is produced upon hydrolysis of PI(4,5)P_2_. IP3R-mediated release of Ca^2+^ from the ER store (store emptying) reduces the luminal ER Ca^2+^ concentration triggering ER store-refilling through *s*tore-*o*perated *Ca*^2+^ *e*ntry (*SOCE*; Smyth et al., 2010; Vaca, 2010). During SOCE, the apposition of ER tubules to the plasma membrane permits ER-resident Ca^2+^ sensor proteins, called *s*tromal *i*nteracting *m*olecules (*STIMs*), to directly bind and activate plasma membrane-resident Orai Ca^2+^influx channels (Park et al., 2009). Employing a variety of experimental strategies, we found that productive virus assembly and IP3R function were directly correlated. Agonists known to promote PI(4,5)P_2_-dependent Ca^2+^ signaling stimulated Gag accumulation on the plasma membrane and budding of the assembled Gag particles while antagonists that interfered with signaling inhibited both (Table 1). Some requirements, e.g., for Gαq and events linked to SOCE, became apparent following disruption of the PTAP Late (L) domain. The PTAP L domain serves as docking site for the cellular protein, Tsg101, which is required for efficient virus particle budding (Garrus et al., 2001; Martin-Serrano et al., 2001; VerPlank et al., 2001). Here, we show that modulation of ER Ca^2+^ release and ER store refilling are components of productive Gag assembly and that these events are facilitated by recruitment of Tsg101 by the PTAP L domain in Gag.

**Table 1:** **Observations Linking HIV-1 Budding to PI(4,5)P_2_-PLC-IP3-IP3R-Ca^2+^ Signaling Cascade**.

**Agonists that promote virus budding**
Extracellular Ca^2+^/Ionomycin-induced rise in cytosolic Ca^2+ ^a^^,^^b^^,^^c^^
Thapsigargin-induced ER store depletion ^a^
IP3R-FLAG adventitious expression ^a^
**Antagonists that inhibit virus budding**
5-phosphatase IV-induced PI(4,5)P2 depletion from plasma membrane ^d^^,^^e^
U73122-mediated inhibition of PLC activity ^a^
High affinity-binding IP3R fragment-induced sequestration of IP3 ^a^
Targeted antibody blockade of IP3 binding ^a^
siRNA-mediated IP3R depletion ^a^
siRNA-mediated PLCγ depletion ^f^

a Ehrlich et al., 2010;

b Perlman and Resh, 2006;

c Grigorov et al., 2006;

d Fernandes et al., 2011;

e Ono et al., 2004;

f *Ehrlich et al., 2011*.

## Materials and methods

### Plasmids and antibodies

Plasmids encoding pCMV-Gag-EGFP (WT HIV-1 Gag C-terminally tagged with green fluorescent protein, Hermida-Matsumoto and Resh, 2000), WT Gag-HA and Δp6-Gag-HA (Jin et al., 2007) were kind gifts of M. Resh and J. Jin, respectively. Plasmids encoding P7L-Gag-GFP, Y36S-Gag-GFP, and P7L/Y36S-Gag-GFP were engineered by site-directed mutagenesis using Gag-GFP and P7L-Gag-GFP as templates (Medina et al., 2011). Primary antibodies were: polyclonal rabbit anti-HIV-1 CA (Ehrlich et al., 1992); anti-HIV-1 p6 (a kind gift of S. Campbell); anti-IP3R type 1 (Affinity BioReagents); anti-IP3R type 3 (BD Biosciences); anti-p85 α-subunit of phosphatidylinositol 3-kinase (PI3K, Santa Cruz Biotechnology, Inc.); mouse monoclonal anti-PI(4,5)P2 (Abcam); rabbit anti-IP3R type 2 and anti-actin (Sigma). Secondary antibodies were obtained from: goat anti-rabbit IgG IRDye 800 (Rockland); goat anti-rabbit IgG IRDye 680LT (LI-COR); goat anti-mouse IgG Alexa Fluor 680; TRITC-tagged secondary IgG (Molecular Probes); and gold-conjugated donkey anti-rabbit IgG (Jackson Immuno Res Labs).

### Cell culture, transfection, and harvest

COS-1 and COS A2.5 (Babé et al., 1995) cell culture, DNA transfection using XtremeGene reagent (Roche) and harvest of cells and tissue culture media were done as previously described (Goff et al., 2003; Ehrlich et al., 2011). For 2-aminoethoxydiphenyl borate (2-APB, Calbiochem) treatment: Stock solutions were prepared using dimethyl sulfoxide (DMSO) as solvent. Treatment media with DMSO only or with 2-APB at 50, 100, and 150 μM final concentration in Ca^2+^-free DMEM was prepared fresh prior to use. At 24 h post-transfection, the tissue culture media was aspirated and replaced with treatment media and the plates returned to the incubator. After 24 h, tissue culture media was collected, passed through a 0.45 μm filter for use in VLP isolation and the cells were scraped and pelleted for use in preparing cell lysates.

### Protein delivery with bioporter reagent

COS-1 cells were grown on coverslips in 6-well plates. For early antibody delivery, media was aspirated and replaced with 500 ul of DMEM and delivery mixture (antibody mixed with the BioPORTER reagent, Genlantis) prepared according to manufacturer instructions was added dropwise to the cells. After 4 h in the 37°C incubator, the delivery mixture was removed, replaced with 2 ml of fresh complete media and the cells transfected with DNA encoding Gag-GFP following the usual transfection protocol. Following an additional 24 h incubation period, the coverslips were processed for analysis by deconvolution confocal microscopy. For late antibody delivery, the cells were transfected with DNA encoding Gag-GFP following the usual protocol. After 24 h of incubation, tissue culture media was aspirated, replaced by 500 ul of DMEM and freshly prepared delivery mixture was added drop-wise. At the end of 4 h, the delivery mixture was removed and replaced with 2 ml of fresh complete media. Following an additional 24 h incubation, the coverslips were processed for deconvolution confocal microscopy. Cells on slides were fixed in 3.7% formaldehyde, permeabilized with 0.1% Triton X-100, blocked with 1% BSA, incubated with TRITC (*red*)-tagged secondary antibody and the nucleus stained with DAPI (Molecular Probes). Slides were mounted and fluorescent signals viewed and captured with an inverted fluorescent/dic Zeiss Axiovert 200 M microscope operated using Axiovision software.

### Ca^2+^ measurements

Intracellular free Ca^2+^ ion was determined in cultures of cells that were mock-transfected cells, transfected with WT Gag or the Δp6Gag mutant as previously described (Guo et al., 2005). Briefly, cells were detached with a buffer (Hank's Buffered Salt Solution, HBSS) stream, counted, incubated with Fura-2 AM (Sigma), pelleted, washed, and resuspended in HBSS, with or without EGTA. Measurements were done at room temperature in an ISS spectrofluorometer with continuous stirring of the sample. Samples were excited at 340 and 380 nm and fluorescent signals emitted at 510 nm were recorded at intervals over a 2 min period. The ratio of fluorescence emitted at 340 and 380 nm was converted to free calcium ion concentration [Ca^2+^](nM).

### Sucrose gradient floatation

Pelleted cells were washed three times with cold PBS, swollen in 1 ml of cold hypotonic buffer (10 mM Tris, pH 7.4, 1 mM MgCl_2_) containing protease inhibitors and disrupted with a Dounce homogenizer. The homogenate was spun for 10 min at 1000 × g to obtain a post-nuclear supernatant which was subsequently centrifuged at 27,000 × g to collect a membrane-rich pellet (P2). The P2 pellet was resuspended in 200 μl of a 40% sucrose solution (w/v in PBS), placed at the bottom of the centrifuge tube, and overlaid step-wise with 200 μl of 40, 30, 20, and 10% sucrose solutions. The samples were centrifuged at 72,000 rpm in a TLA-100 rotor for 60 min. All centrifugations were done at 4°C. Aliquots of the floatation gradient were collected from the top of the centrifuge tube.

### Western analysis

Proteins were separated by electrophoresis in 10% (unless otherwise stated in the text) SDS-polyacrylamide gels and electroblotted onto nitrocellulose membrane or dot-blotted. Following incubation with appropriate primary and secondary antibodies, protein bands were visualized and quantitated (where needed) using an infrared-based imaging system (Odyssey, LI-COR Biosciences). Release efficiency was defined as the ratio of the signal intensity value for the VLP-associated Gag to the sum of the values for VLP-associated Gag plus cell lysate-associated Gag (VLP/[VLP + Cell Lysate]).

### Immuno-electron microscopy

Cells grown on ACLAR film were fixed in 4% paraformaldehyde/0.1% EM grade glutaraldehyde (Electron Microscopy Sciences) in PBS and processed for resin embedding following standard protocols. The thin sections were incubated with rabbit anti-IP3R type3 polyclonal antibody and subsequently with gold-conjugated donkey anti-rabbit antibody. Sections were counterstained with uranyl acetate and lead citrate and viewed with a FEI Tecanal BioTwinG2 electron microscope.

### Deconvolution confocal microscopy

Cells on coverslips were fixed in 3.7% formaldehyde (Fisher) for 20 min, and then permeabilized in 0.1% Triton X-100. All images were captured on an inverted fluorescence/differential-interference contrast Zeiss Axiovert 200 M deconvoluting fluorescence microscope operated by AxioVision version 4.5 (Zeiss) software. Ten to 20 optimal sections along the *z* axis were acquired in increments of 0.4 μm. The fluorescence data sets were deconvoluted by using the constrained iterative method (AxioVision). Images shown are of the central focal plane unless otherwise stated. To quantify relative co-localization of signal from two (*red and green*) channels, Pearson correlation coefficients were obtained using Image J quantification software down-loaded from NIH website http://rsbweb.nih.gov/ij/index.html. Co-localization analysis was performed using the co-localization finder plug-in. The significance level of correlation coefficients was assessed by reference to http://www.jeremymiles.co.uk/misc/tables/pearson.html.

### Total internal reflection fluorescence (TIRF) microscopy

COS-1 cells were grown on 25 mm cover slips coated with poly-lysine and co-transfected with WT Gag-GFP and SS KDEL-RFP plasmids. Cell imaging was done after a 20 h incubation period. Immediately prior to imaging, the cover slip was rinsed in Imaging Buffer (130 mM NaCl, 2.8 mM KCl, 5 mM CaCl_2_, 1 mM MgCl_2_, 1 mM NaH_2_PO_4_, 10 mM HEPES, pH 7.4), then mounted in an open bath chamber with ~1 mL of Imaging Buffer. Cells were imaged with an Olympus IX-81 microscope equipped with an Olympus TIRF Launch, Olympus 60× Plan Apo, *N* = 1.45 TIRF objective, 2× optovar, Photometrics DV2 dual-view image splitter, and Andor iXon CCD camera. Fluorescent proteins were excited with Olympus Cell* digital lasers with AOTF shutters at 488 and 561 nm. The objective was equipped with a Semrock LF488/561-A-000 filter cube, with 482/563 excitation filter, 523/610 emission filter, and 488/561 dichroic mirror. The dual-view was equipped with Chroma 11-EM GFP/RFP (565 dcxr) filter cube, with D520/30 and D630/50 m emission filters. The TIRF angle and laser AOTF shutters were controlled with the native Olympus Cell^TIRF software, and images were recorded with Metamorph Premier (Molecular Devices) software. Image frames were acquired with alternating 488 and 561 nm excitation, with 100 ms exposures at 2 Hz. For image analysis, the red and green channels of cell images were aligned using the calculated alignment of an image of Fluospheres 505/515 (Invitrogen) yellow-green emitting, 100 nm polystyrene beads captured immediately prior cell imaging and aligned using in-house Matlab-based software (Mathworks). Aligned red and green images were overlaid in Metamorph.

## Results

### Cells expressing Gag exhibit higher cytosolic [Ca^2+^]_*i*_, derived from intracellular stores

**Figure 2:**
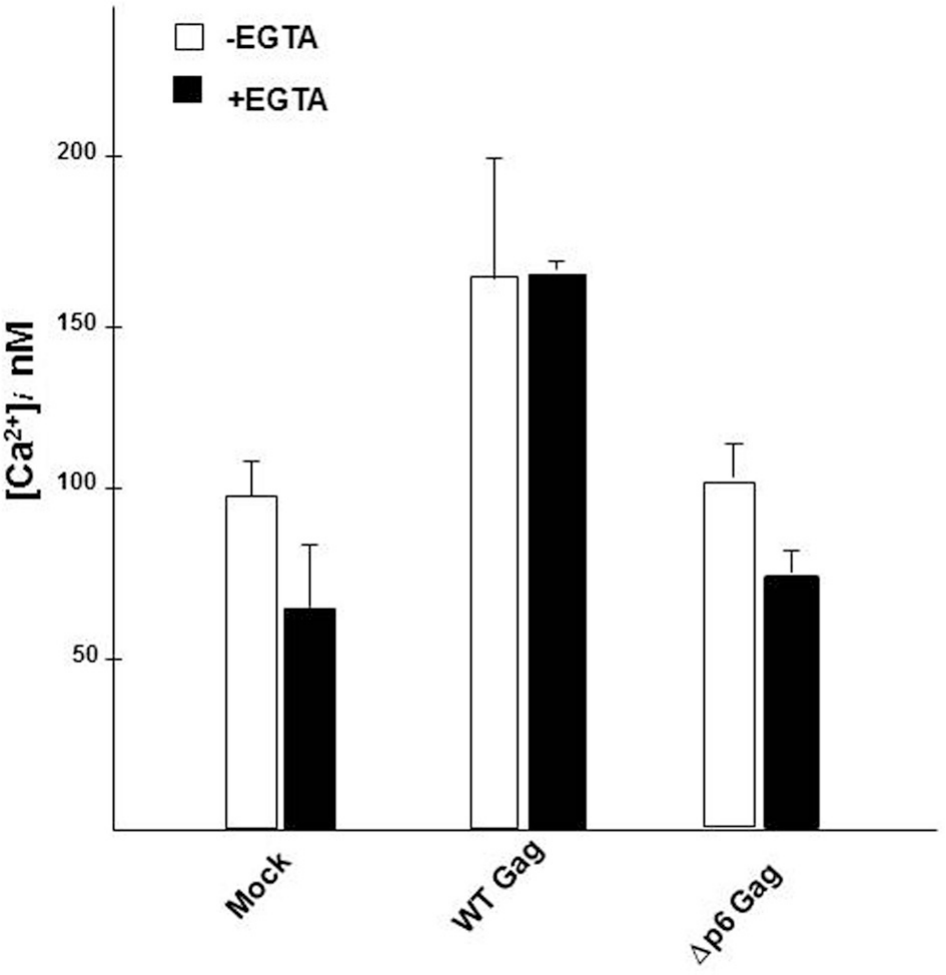
**Gag expression induced elevation of cytosolic Ca^2+^ concentration**. Mock-transfected cultures of COS cells or cultures transfected with WT Gag or Δp6 Gag were assayed for Ca^2+^ in the absence (*open symbol*) or presence (*filled symbol*) of EGTA. Each bar represents the average value determined for [Ca^2+^]_*i*_ in 3 independent trials using triplicate samples. In each trial, [Ca^2+^]_*i*_ was assayed every 6 s over a 2 min period. The standard error of the mean was ±3% for the mock-treated samples and ±5% for the *gag*-transfected samples.

The concentration of free, unbound Ca^2+^ ion in the cytosol of the cell ([Ca^2+^]_*i*_) is maintained at low levels (~100 nM) due to the combined action of the binding of the Ca^2+^ ions to buffers and protein adaptors, its translocation into intracellular stores and its extrusion out of the cell (Berridge et al., 2003; Hanson et al., 2004; Clapham, 2007). We (Ehrlich et al., 2011) previously provided evidence that [Ca^2+^]_*i*_ in cells expressing WT Gag was higher than in mock-treated cells or in cells expressing a budding-defective Gag mutant. The mutant, P7L-Gag, possesses a single residue change in the primary L domain (P_7_TAP to L_7_TAP) that impairs Tsg101 binding to the site (Demirov et al., 2002). That earlier study, where we utilized a cell imaging-based assay for measuring free unbound Ca^2+^ ions in the cytosol, indicated that Gag expression was accompanied by a significant increase (~1.5-fold) in [Ca^2+^]_*i*_. Here, we confirm that finding, using an assay that examines the entire population of transfected cells (Figure 2). A 1.5-fold increase in [Ca^2+^]_*i*_ was observed in cells that had been transfected with DNA encoding WT Gag over the level measured for cells expressing Δp6 Gag, a mutant missing PTAP and the other L domains, and over the level obtained for mock-transfected cells. Detection of this difference in the assay of the culture indicates that most of the cells in the culture underwent the change. Moreover, as was the case in the single cell imaging-assay, the higher [Ca^2+^]_*i*_ was observed in the presence or absence of 2 mM EGTA, a cell-impermeant chelator of Ca^2+^ ions, indicating that the increase in [Ca^2+^]_*i*_ did not require influx of the ion from the extracellular environment. The results indicate that (i), Gag expression leads to an increase in cytosolic Ca^2+^ through release of the ion from intracellular stores; (ii), the L domains housed in the p6 region of Gag are determinants of the increase and (iii), this change occurred in a large number of the cells in the culture.

### IP3R activation is required throughout the assembly period to retain Gag at the plasma membrane

**Figure 3:**
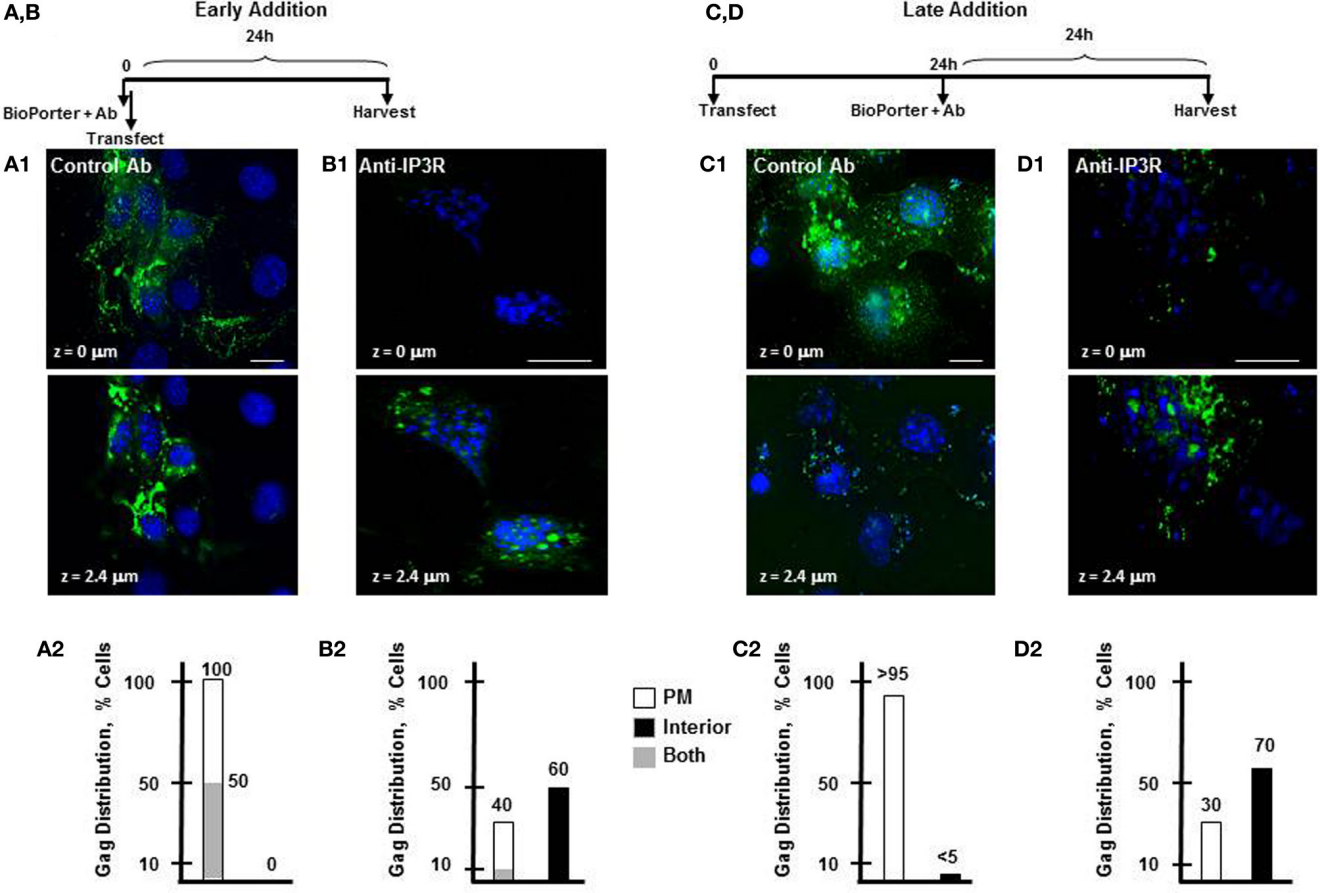
**Interference with IP3R function prevents Gag accumulation on the plasma membrane. (A,B and C,D)** Schematic diagram of protocol for early **(A,B)** and late **(C,D)** delivery of anti-IP3R antibody. **(A1)** Control antibody, or **(B1)**, anti-IP3R rabbit antibody directed to the highly conserved IP3 binding domain on IP3R were delivered into cells 4 h prior to transfection of DNA encoding Gag-GFP. Twenty-four hours later, the cells were prepared for deconvolution confocal microscopy. **(C1,D1)** Cells were first transfected with DNA encoding Gag-GFP DNA and after 24 h, subjected to protein transduction of control antibody **(C1)** or the anti-IP3R antibody **(D1)**. After an additional 24 h incubation, cells were fixed and processed as above. **(A2–D2)** Quantitative analysis of cells containing Gag at the plasma membrane exclusively (*open bars*), in the cell interior exclusively (*filled bars*), or in both locations (*gray bars*), based on examination of 80–100 cells, *n* = 2. Dapi stain (*blue*) was used to label the nucleus. The figure shows sections through the z plane of cells representing the plasma membrane-proximal (*z* = 0) and the cell interior (*z* = 2.4). Bars indicate 10 μm.

ER store Ca^2+^ that is released to the cytosol is rapidly taken up by cellular Ca^2+^ binding buffers and proteins (Schwaller, 2010; Yanez et al., 2012). Our finding that [Ca^2+^]_*i*_ in Gag-expressing cells is above basal level at steady state indicates that Gag assembly induces Ca^2+^ store release events to occur in the cell. We had previously shown that IP3R is required for Gag association with the plasma membrane (Ehrlich et al., 2010). We therefore asked whether IP3R mediation of Ca^2+^ release from the ER store is required for accumulation of Gag at the plasma membrane as well. To test this, we employed the BioPORTER reagent as a protein delivery vehicle to introduce anti-IP3R antibody targeted specifically to the activation domain of IP3R either simultaneously with DNA transfection or 24 h later (Figure 3, early or late addition, *respectively*). To account for possible non-specific effects, control antibody (polyclonal antibody directed at the p85 α-subunit of PI3K) was delivered to transfected cells. We have used both of these anti-IP3R and control antibodies in microinjection experiments where each was co-injected with Gag-encoding DNA into cells that were subsequently examined by deconvolution confocal microscopy and have demonstrated that anti-IP3R antibody, but not control antibody, prevented association of Gag at the plasma membrane (Ehrlich et al., 2010). In the current experiment, COS-1 cells grown on coverslips and transfected with Gag-encoding DNA at *T* = 0 were treated with an antibody-BioPORTER mixture either immediately or 24 h later. Thus, for the early addition protocol, the coverslips were processed for deconvolution confocal microscopy at 24 h post-DNA transfection; for the late addition protocol, samples were processed at 48 h post-DNA transfection. The early interference protocol control samples (**A1** panels) indicated that Gag was detected at the plasma membrane in 100% of cells examined (100/100, panel **A2**). In half of these cells, Gag was additionally in the cell interior (panel **A1** top, cell periphery, *z* = 0 μm; panel **A1** bottom, cell interior, *z* = 2.4 μm). Comparable results were obtained in the transfected cells that received buffer only (i.e., mock-treated; *not shown*). That Gag was mostly at the plasma membrane is consistent with the known rapid trafficking of Gag to the plasma membrane and its primary localization to that membrane compartment at steady state (Hermida-Matsumoto and Resh, 2000; Jouvenet et al., 2006; Perlman and Resh, 2006). In cells receiving anti-IP3R antibody (**B1** panels), Gag was mainly detected in the cell interior z section (panel **B1**, bottom, *z* = 2.4) in contrast to the results obtained in the control sample, where all cells had Gag at the cell periphery (panel **A1**, top, *z* = 0). Analysis indicated that Gag was detectable at the plasma membrane in only 40% of cells examined (32/80, panel **B2**) with the remainder of the cells showing Gag exclusively in the perinuclear region in contrast to the control. In some cases, Gag was detected in round intracellular vacuoles, reminiscent of the results obtained in previous studies where immuno-gold tagged Gag was found to accumulate in 200–500 nm vesicles in the cell interior following siRNA-mediated IP3R depletion (Ehrlich et al., 2010). The late interference protocol control samples (**C1** panels) showed Gag at the plasma membrane in essentially all cells examined (95/100, panel **C2**, *z* = 0). In contrast, in cells receiving anti-IP3R antibody (**D1** panels) Gag was detected mainly in the cell interior z section (panel **D1**, bottom, *z* = 2.4). Analysis revealed Gag at the plasma membrane only in 30% of cells examined (30/100, panel **D2**) and mainly in the interior in the other cells. This result indicates that anti-IP3R delivery at 24 h post DNA transfection caused the Gag molecules that were already at the plasma membrane at this time (see **A1** panels) to be removed from the plasma membrane. We conclude that IP3R activation is continuously required for association and retention of the Gag form that was on the plasma membrane. This provides a possible explanation for the elevation in cytosolic Ca^2+^ detected in cells supporting productive Gag assembly.

### ER and Gag are in proximity at the cell periphery

**Figure 4:**
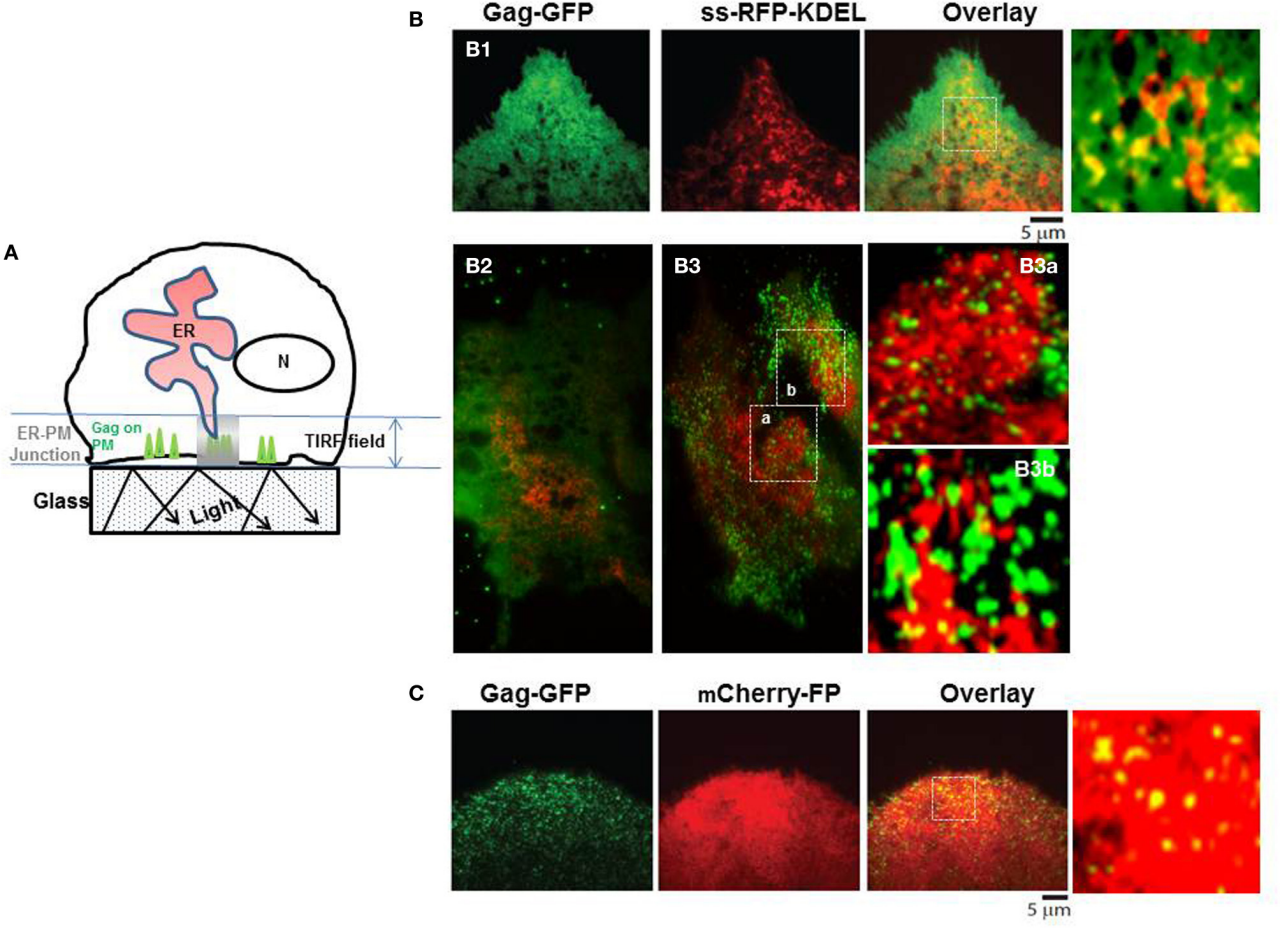
**TIRF microscopy of peripheral ER in proximity to Gag at the plasma membrane. (A)** Schematic drawing depicting area of signal capture in TIRF field. Panels **(B,C)** show representative images of Gag-GFP fluorescence (green) at an early (8 h, **B1**) and a later (24 h, **B2,B3,C)** time point. SS-KDEL-RFP (*red*) labels the ER **(B)**; mCherry-FP (red) labels the cytosol **(C)**. Indicated regions (white box) in **(B,C)** are enlarged in **(B1,B3,C)**. Overlay shows that some puncta are yellow, indicating co-localization of red and green signals.

The higher cytosolic Ca^2+^ in WT Gag expressing cells, whether measured in single cells (Ehrlich et al., 2011) or in the cell culture (cf. Figure 2) suggests an L domain-mediated modulation of store Ca^2+^ release activity. By analogy to proteins that have been shown to influence IP3R channel activity such as RACK1 (Patterson et al., 2004b) or 80K-H (Kawaai et al., 2009), the ability to influence IP3R channel activity infers physical proximity of Gag to IP3R. Although we have not observed Gag co-localization with the ER body in the cell interior, we previously showed that Gag co-localizes with FLAG-tagged IP3R on the plasma membrane and in ER-derived tubules near the cell periphery (Ehrlich et al., 2011), providing opportunity for proximity to ER store-released Ca^2+^. As it is generally recognized that ER-plasma membrane (ER-PM) junctions, i.e., dynamically formed regions where the ER is in close apposition to the plasma membrane, are a feature of all eukaryotic cells (Friedman and Voeltz, 2011), we examined cells by *t*otal *i*nternal *r*eflection *f*luorescence (*TIRF*) to determine whether the peripheral ER, revealed by the luminal marker ss-RFP-KDEL, was sufficiently close to plasma membrane-localized Gag-GFP to make feasible a functional link with IP3R channels on ER membrane (Figure 4). TIRF (reviewed in Steyer and Almers, 2001) allows visualization of only those fluorescent signals near the plasma membrane where signal is generated only in fluorophores that are positioned in the near plasma membrane region (i.e., from the coverslip surface to ~100 nm above the coverslip) as illustrated in the cartoon (panel **A**). The images (panel **B**) revealed that fluorescent signals were evident in the TIRF field from both the Gag-GFP on the plasma membrane (*green*) and the ER lumen marker ss-RFP-KDEL (*red*) whether cells were examined at 8 h post-transfection (panel **B1**) or 24 h post-transfection (panels **B2,B3**). Consistent with previous studies (Jouvenet et al., 2008), Gag was detected both as a diffuse signal and as discrete puncta. The latter were previously demonstrated to exhibit the characteristics expected of genuine higher-ordered Gag assemblages or VLPs while the former were suggested to be low oligomeric Gag forms (Jouvenet et al., 2008). As expected based on previous studies (Jouvenet et al., 2008), at the early time Gag exhibited a diffuse distribution; later both diffuse and punctate signals were detected. At 24 h post-transfection, the majority of cells are expected to exhibit a predominantly punctate signal as shown in panel **(B3)**; an image from a cell exhibiting fewer puncta (panel **B2**) was included to show that both low oligomer and higher-ordered Gag assemblages were detected in the region. Control studies using mCherry-FP as a marker for cytosol (panel **C**) revealed co-localization of Gag with this marker, indicating that cytosol components also were accessible to Gag in the ER-PM junctional region. This is as expected since Ca^2+^ is released into this space. At both times post-transfection, image overlays revealed yellow puncta, indicating sites of red and green signals in very close proximity suggesting the presence of assembling Gag and VLPs in the plasma membrane regions encompassed by the ER-PM junctions. It should be noted that ER-PM junctions are detectable in most cells (Friedman and Voeltz, 2011), making it difficult to ascertain whether Gag possesses any inducing effect on junction formation. Nevertheless, ER-PM junctions present themselves as sites where IP3R channels on tips of peripheral ER can conceivably get within interaction distance of plasma membrane-localized Gag molecules.

**Figure 5:**
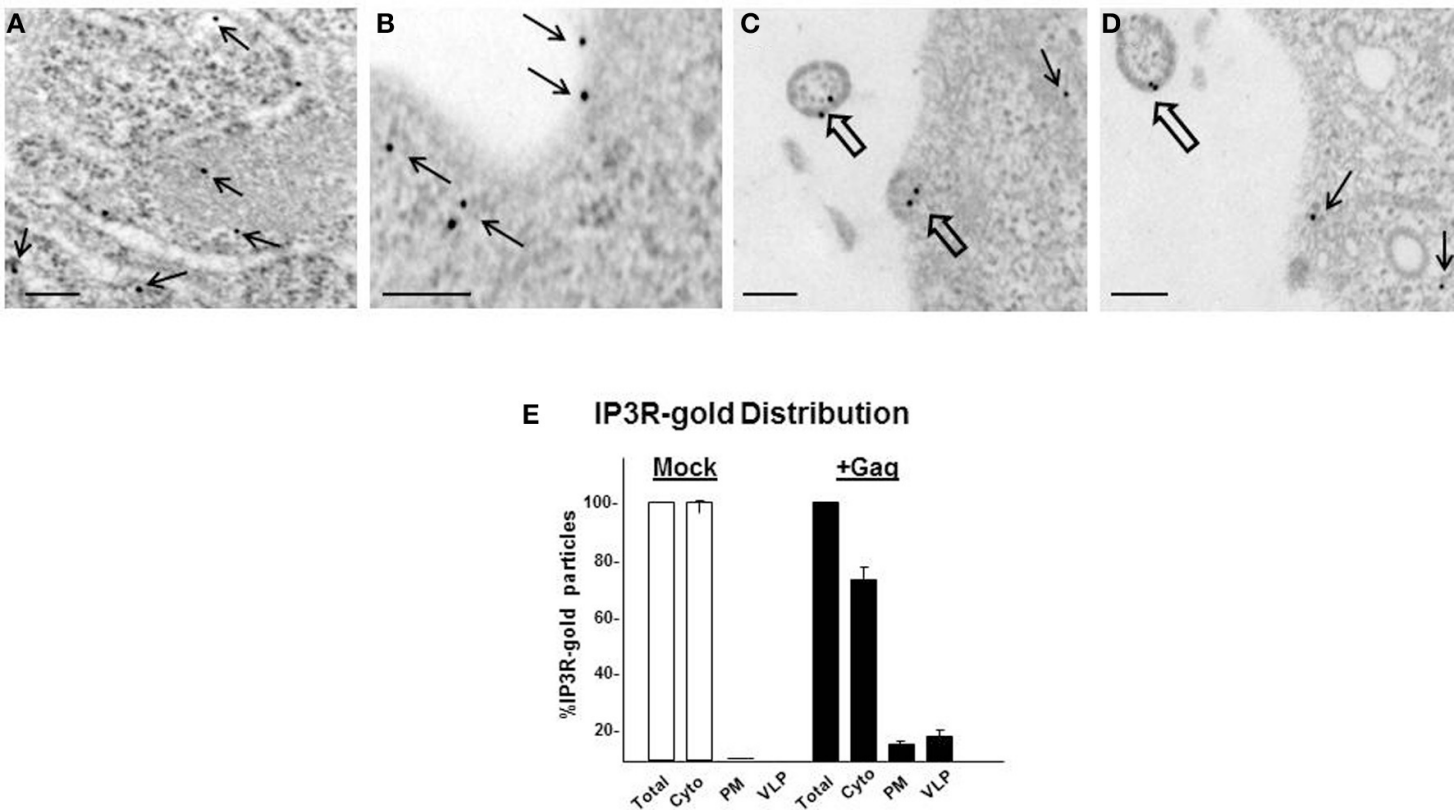
**Gag and IP3R are in proximity at the plasma membrane. (A)** Immunogold labeling of endogenous IP3R-3 in mock-transfected cells. **(B–D)** Cells transfected with DNA encoding WT Gag. Panels show representative images with IP3R-tagged gold detected at or near the plasma membrane (*thin arrows*) and in budding or fully released VLPs (*thick arrows*). Bar: 100 nm. **(E)** Distribution of gold-tagged IP3R in mock-treated cells and cells expressing Gag.

That IP3R can come close to plasma membrane-localized Gag was suggested by our earlier observation that adventitiously expressed IP3R-FLAG co-localized at the cell periphery with WT Gag but, interestingly, not with P7L-Gag (Ehrlich et al., 2011). We wanted to eliminate the possibility that the observed co-localization might be unique to adventitious expression and therefore examined endogenous IP3R for proximity to PM-localized Gag. To assess Gag proximity to endogenous IP3R, we examined mock-treated cells or cells transfected with DNA encoding Gag by immunoelectron microscopy (IEM; Figure 5). Cells grown on ACLAR were transfected with DNA encoding WT Gag and at 36 h post-transfection the ACLAR was processed as described in Materials and Methods and examined for IP3R-tagged gold signal. Cells supporting productive Gag assembly were identified by detection of cell-associated virus-like particles (VLPs). Cells were examined for IP3R-3 using a primary mouse monoclonal antibody that recognized the IP3R isoform and secondary donkey anti-mouse polyclonal antibody tagged with 15 nm gold particles. An average number of 11 (210/19) or 14 (224/16) gold particles was detected in the cytoplasm of mock- or *gag*-transfected cells, *respectively* (*n* = 2). No gold was detected in cells depleted of the IP3R-3 target of the antibody by siRNA-mediated interference prior to *gag*-transfection (*data not shown*). Similarly, gold was not detected when the primary antibody was omitted (*not shown*). In the mock-transfected cells, the gold was almost exclusively found in the cell interior (panel **A**). In contrast, in cells in the *gag*-transfected culture, gold particles were detected at the cell periphery (panel **B**, narrow arrows) and in budding (panel **C**) or released (panel **D**) VLPs (broad arrows). Detection of gold signal on VLPs, suggesting virion-association of IP3R, is consistent with previous proteomic analysis that revealed IP3R encapsidation in purified infectious virus (Chertova et al., 2006). Quantification of the gold-tagged IP3R distribution (panel **E**) indicated that the level of plasma membrane-associated IP3R was >20-fold higher in *gag*-transfected cells (12%) compared to mock-treated cells (0.5%). The level was >50-fold higher if compared to gold particles associated with both the plasma membrane and VLPs. The results indicate a significant re-distribution of IP3R in cells expressing Gag, consistent with our previous findings using FLAG-tagged IP3R (Ehrlich et al., 2011).

**Figure 6:**
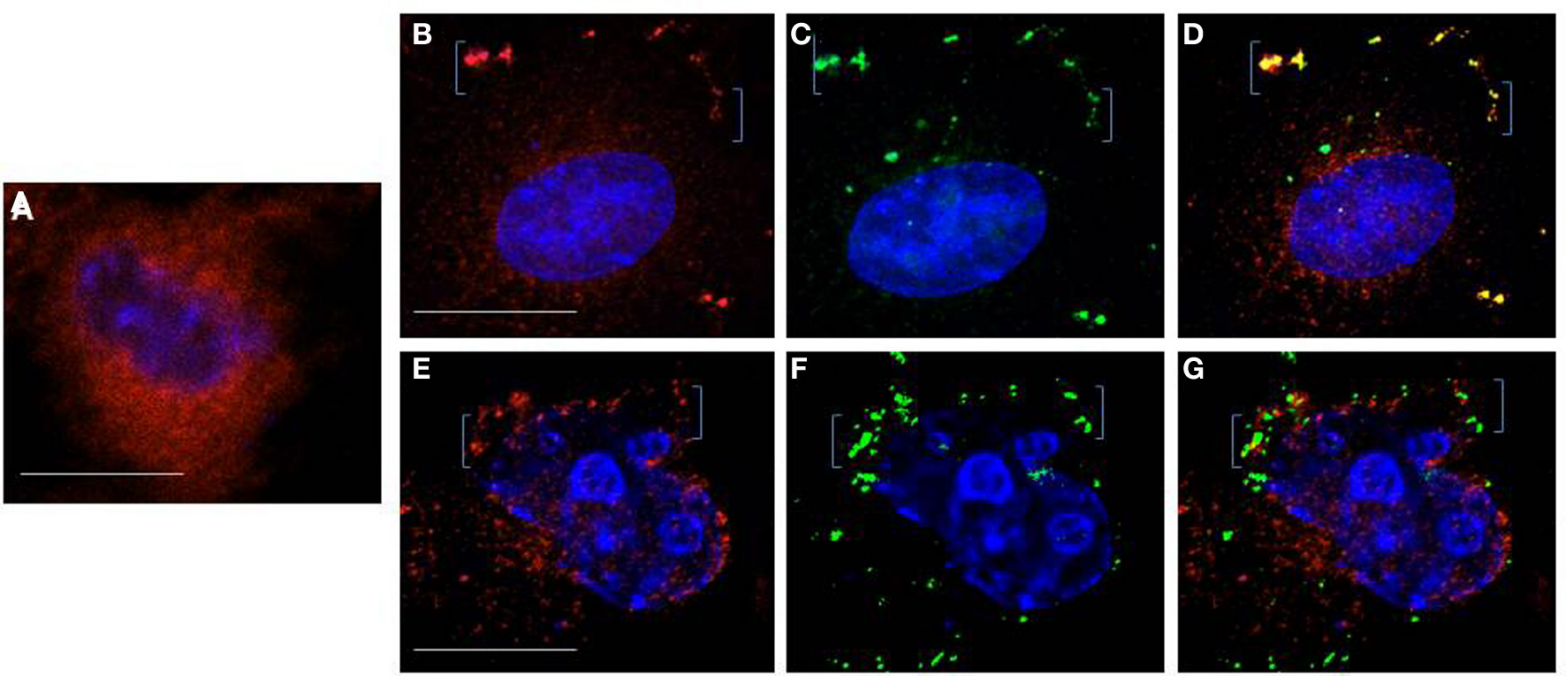
**Immunostaining of IP3R**. Anti-IP3R antibody delivered into cells using Bioporter reagent was used to detect endogenous IP3R in mock-treated cells **(A)** or cells that were transfected with DNA encoding Gag-GFP (**B–D** and **E–G**). Twenty-four hours post-transfection, cells were fixed, permeabilized, incubated with TRITC-(*red*)-tagged secondary antibody and examined by deconvolution confocal microscopy to visualize IP3R (*red*) and Gag-GFP (*green*). Brackets indicate regions of Gag-IP3R proximity at the plasma membrane. Bar: 10 μm.

As described above, interference with IP3R function by introduction of antibody targeted to its activation site prevented Gag accumulation on the plasma membrane in a majority of cells whether the blockade occurred early or later in the Gag assembly period (cf. Figures 3B2,D2). TIRF analysis detected Gag at ER-PM junctions early and later (cf. Figure 4). As a further test for Gag-IP3R proximity, we examined cells by confocal microscopy. Anti-IP3R antibody was delivered into mock- or DNA-transfected cells, cells were fixed, permeabilized as described in the legend to Figure 3, and then incubated with a fluorescently-tagged secondary antibody prior to examination by confocal microscopy (Figure 6). While the IP3R signal (*red*) in mock-transfected cells was diffusely distributed in the perinuclear region or throughout the cell interior (panel **A**) as previously reported by our laboratory and others (Vermassen et al., 2004; Ehrlich et al., 2011), the cells in the *gag*-transfected culture exhibited a more punctate signal (panels **B,D**) with some of the signal detected at the cell periphery (bracketed, panels **B,E**). As in TIRF, in some cases, an overlay of the punctate Gag (*green*) and IP3R (*red*) signals produced yellow, suggesting that the proteins co-localized (panels **B–D**, 16/50 cells = 32%, Pearson's coefficient of correlation = 0.7). In other instances, close apposition but not co-localization was observed (panels **E–G**). In either case, these findings are consistent with the results of TIRF and IEM and support the conclusion that Gag and IP3R are in proximity at the cell periphery.

### IP3R and Gag co-immunoprecipitate

**Figure 7:**
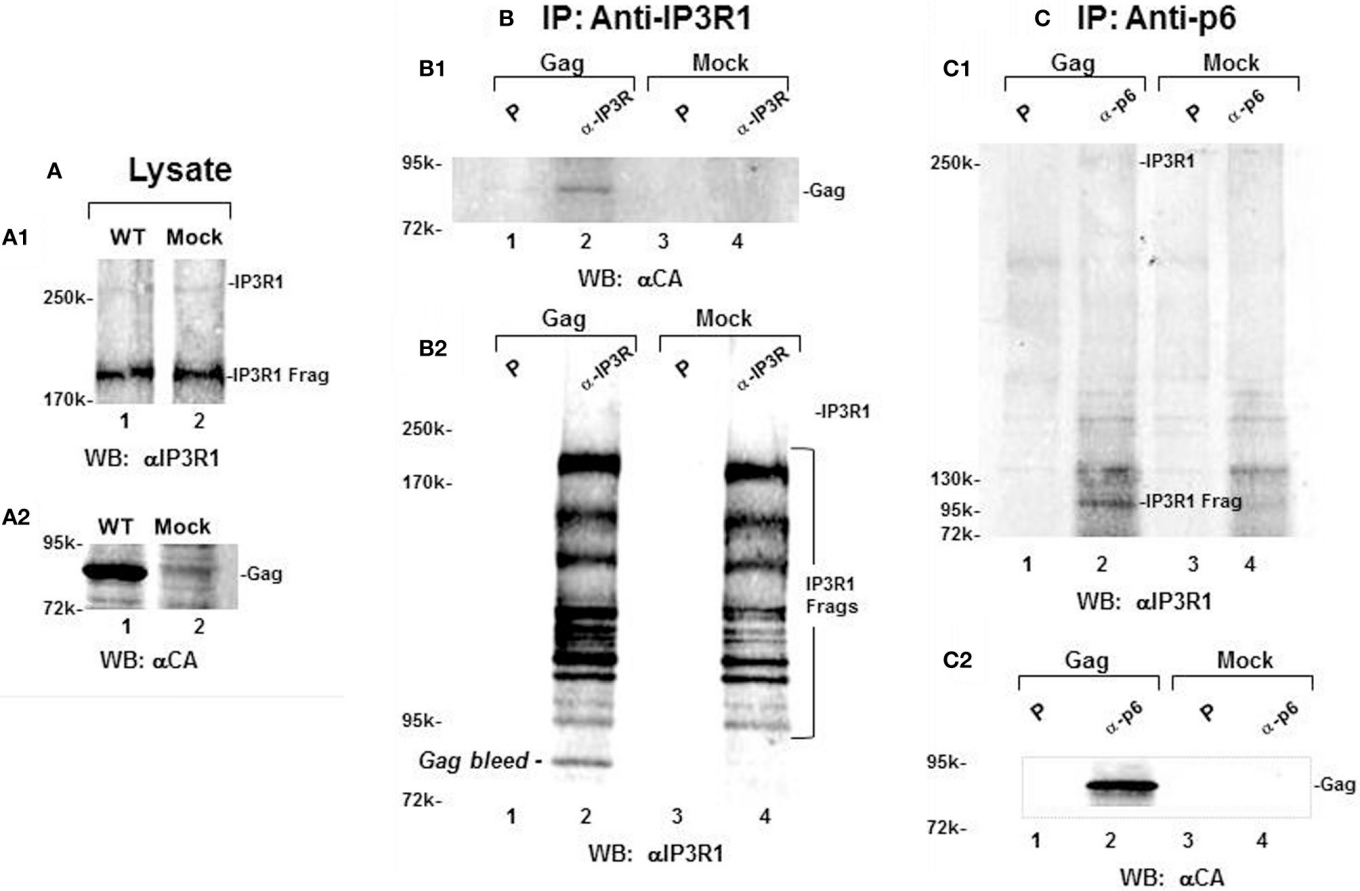
**Gag and IP3R can be reciprocally immunoprecipitated. (A)** Total lysate from cells transfected with Gag (*lane 1*) or from mock-transfected cells (*lane 2*) probed for IP3R (**A1**, *top*) and Gag (**A2**, *bottom*). **(B,C)** Immunoprecipitation of Gag by anti-IP3R antibody **(B)** or by anti-p6 antibody **(C)**. Total lysates were mixed with either preimmune serum (P, *lanes 1,3*) or anti-IP3R antibody (*lanes 2,4*) and immune precipitates were analyzed by Western blotting for IP3R-1 **(B2,C1)** and for Gag **(B1,C2)**. Reciprocal pulldown is shown for Gag by anti-IP3R1(**B1**, *lane 2*) and for a 100 kDa IP3R-related fragment by anti-p6 antibody (**C1**, *lane 2*).

We determined whether the apparent proximity of Gag and IP3R at ER-PM junctions permitted their coimmunoprecipitation from cell lysates prepared by Dounce homogenization of cells in the presence of 1% Triton X-100. Most of the IP3R in such lysates can be expected to derive from the ER body and the network of ER tubules. Cells transfected with DNA encoding WT Gag and mock-transfected cells were Dounce homogenized in lysis buffer containing 1% Triton X-100, clarified by centrifugation at 1000 × g and incubated with pre-immune serum or antibodies that recognized IP3R-1 or Gag (Figure 7). Analysis of lysate (panel **A**) indicated that both mock and transfected samples possessed equivalent amounts of full-length and IP3R fragments that were recognized by an antibody targeted to an epitope at aa1829–1848 (panel **A1**). In contrast, the anti-CA antibody recognized Gag in the lysate prepared from transfected cells but not from the mock-treated cells (panel **A2**). Anti-IP3R1 antibody pulldown (panel **B**) showed Gag in IP3R1-specific immune-precipitates from the lysate of transfected cells but not from the lysate derived from the mock-transfected cells (panel **B1**). The antibody brought down a nearly identical panel of IP3R-related fragments with major forms >95 kDa (panel **B2**). In the reciprocal pulldown experiment (panel **C**), antibody that recognizes the p6 region in Gag formed an immune precipitate that mostly contained a ~100 kDa IP3R-related fragment (panel **C1**, *lane 2*) and Gag (panel **C2**, *lane 2*). This fragment, which was consistently observed, most likely was derived by truncation of the intact 300 kDa IP3R protein, as the latter was detected in relatively small amounts (cf. Figure 7C1). As the apparently more stable 100 kDa IP3R-related protein was co-precipitated reproducibly, we conclude that, in the cell, Gag was in close proximity to IP3R and the ER Ca^2+^ stores.

### IP3R differentially associates with WT Gag and P7L-Gag

**Figure 8:**
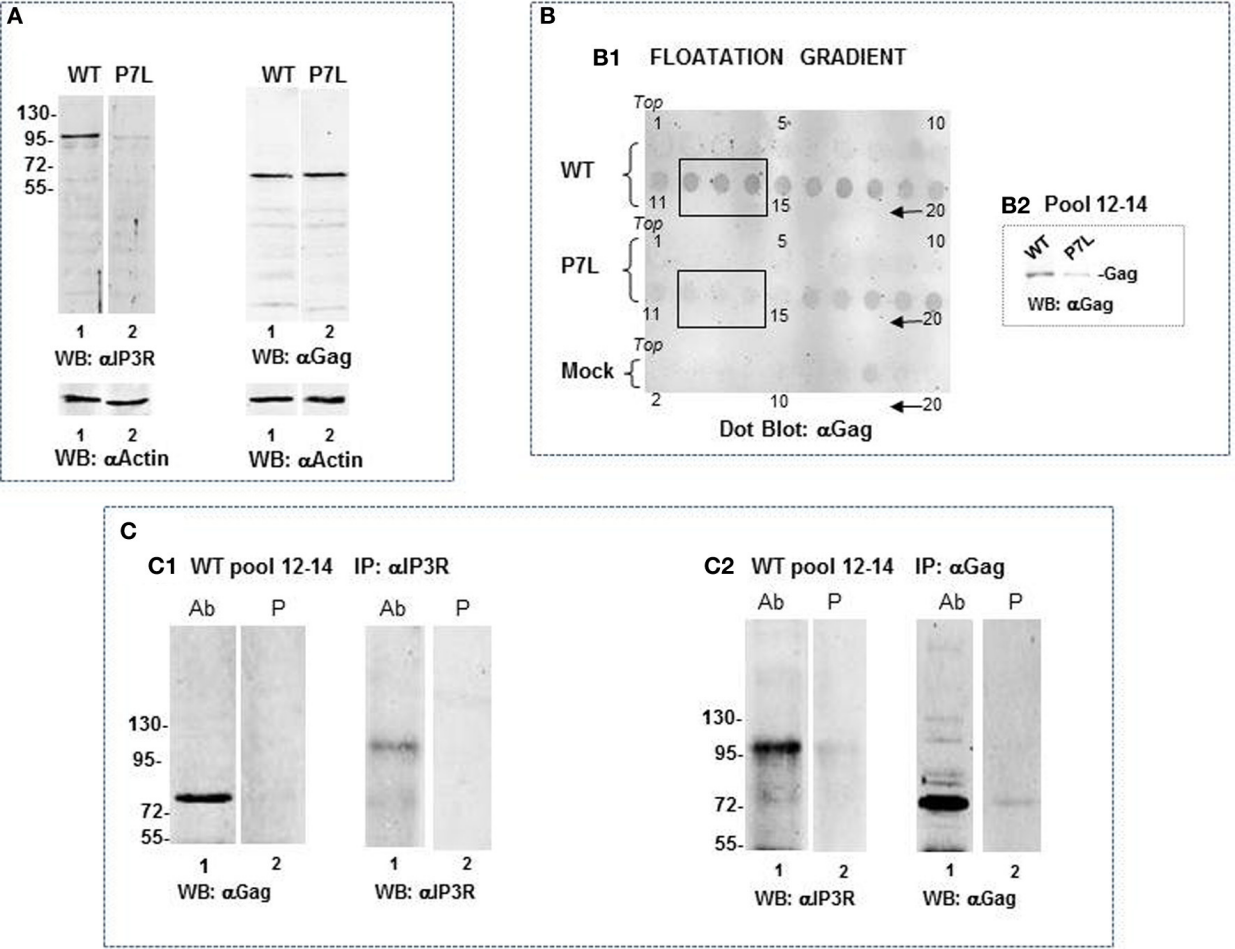
**Differential association of IP3R with WT-Gag and P7L-Gag. (A)** Western analysis of P2 fraction prepared from cells transfected with DNA encoding WT Gag or P7L-Gag. IP3R probing shows ~100 kDa IP3R-related fragment as predominant species detected by antibody. Gag probing shows equivalent amounts of wild-type and mutant Gag in P2 fraction. **(B)** Floatation on sucrose density gradient of P2 fraction prepared from cells that had been transfected with DNA encoding WT Gag (*top*), transfected with P7L-Gag (*middle*), or mock-transfected (*bottom*). Fraction 1 is the top of the gradient. **(B1)** Dot blot analysis of 5 ul of gradient fractions (every fraction for WT and P7L samples and every other fraction for the mock-treated sample). Fractions 12–14 were pooled and analyzed for Gag by standard Western analysis to confirm the presence of Gag and absence of P7L in the light density membranes **(B2)**. **(C)** Co-immunoprecipitation experiment using the pool of fractions 12–14 from WT Gag sample. Western analysis shows detection of Gag in the anti-IP3R1 antibody immunoprecipitate (**C1**, *lane 1*) and detection of the ~100 kDa IP3R-related fragment in the anti-Gag antibody immunoprecipitate (**C2**, *lane 1*).

Because the ~100 k fragment was not well-represented in the total cell lysate as shown in the anti-IP3R1 antibody reprobe (Figure 7B2), we speculated that it might have originated from the ER-PM junction rather than the ER body or ER tubular network. Gag is associated with membranes that should be separable from Golgi and ER by differential centrifugation. To examine this possibility, cells were Dounce-homogenized in the absence of Triton X-100, followed by centrifugation at 1000 × g to obtain a post-nuclear supernate (S1). Further centrifugation of the S1 fraction at 27,000 × g provides a membrane-enriched fraction (P2; Goff et al., 2003). A P2 fraction prepared from cells that had been transfected with DNA encoding WT Gag or P7L-Gag was used to further assess Gag-IP3R subcellular proximity (Figure 8). Panel **(A)** shows a Western analysis of the P2 fraction for IP3R and Gag following solubilization of the fraction in buffer with 1% Triton-X100. In contrast to the total cell lysate in which the anti-IP3R1 antibody detected numerous IP3R fragments (cf. Figure 7B2), the P2 fraction almost exclusively contained the ~100 kDa IP3R-related fragment (panel **A**). Interestingly, the lysates derived from cells expressing WT and P7L-Gag exhibited a clear a difference in the amount of this fragment. To examine the basis for this difference, further separation of membranes in the P2 fraction was conducted through floatation on sucrose. As shown in panel **(B)**, WT Gag and P7L-Gag were both detected in gradient fractions 16–20 but only the WT protein floated to the lighter density region of the gradient (fractions 11–15). This bias in WT Gag floatation was confirmed by Western analysis of pooled fractions 12–14 (panel **B2**). Using the WT Gag-containing pool of fractions 12–14 in immune-precipitation experiments (panel **C**) showed that addition of anti-IP3R antibody pulled down Gag (panel **C1**, *lane 1*) and, reciprocally, addition of anti-Gag antibody pulled down the ~100 kDa IP3R-related fragment (panel **C2**, *lane 1*). Neither pull-down was observed with use of pre-immune serum (*lane 2*). This observation implicates Tsg101 binding in facilitating Gag localization to a membrane context where the Gag and IP3R proteins are in proximity.

### L domain-dependent modulation of store refilling

**Figure 9:**
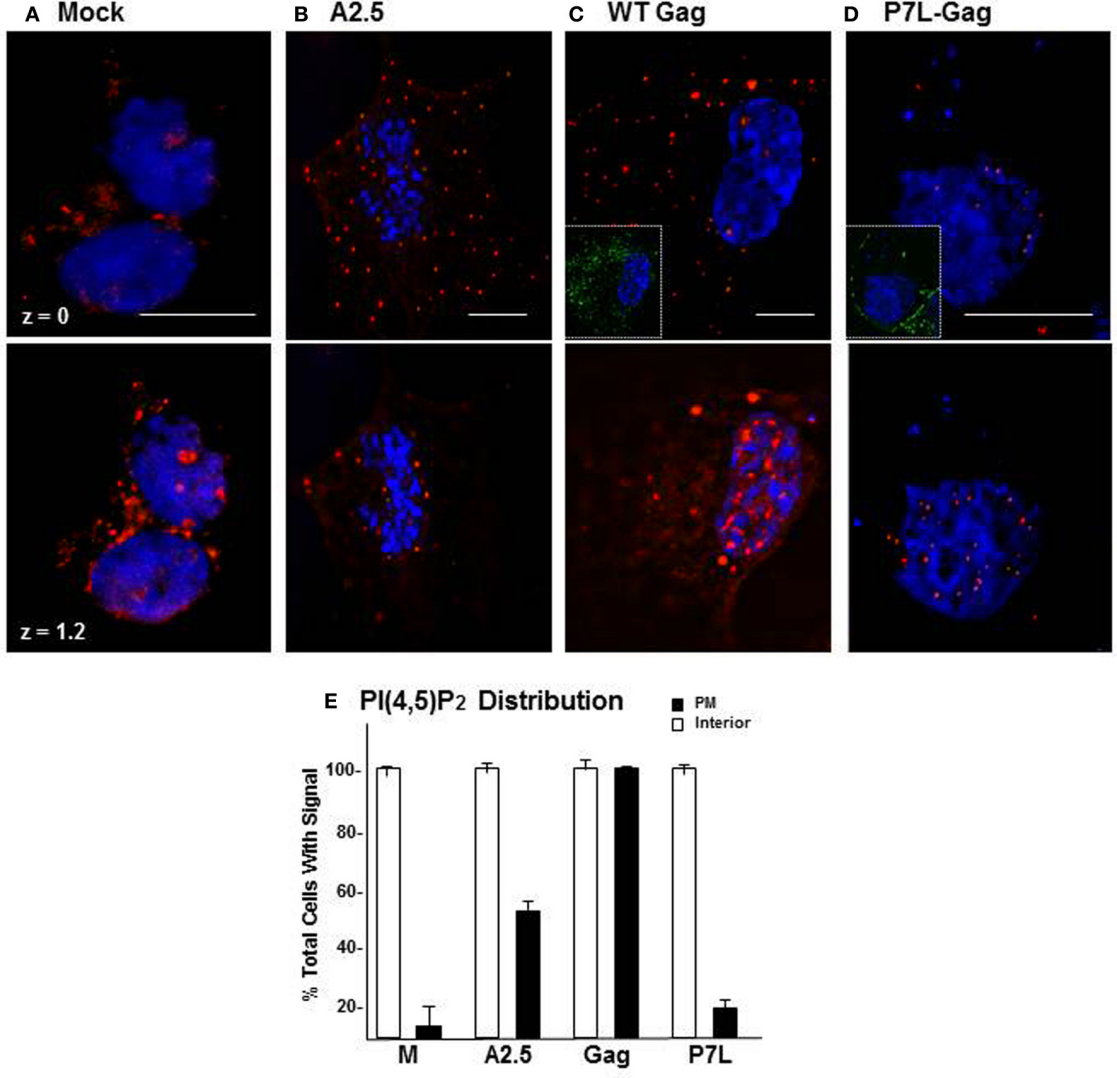
**Accumulation of PI(4,5)P_2_ in Gag expressing cells is L domain dependent**. Mock-treated COS-1 cells **(A)**, COS-12A2 (A2.5) cells that express Gag constitutively **(B)**, or COS-1 cells transfected 24 h with DNA encoding WT Gag **(C)** or P7L-Gag **(D)** were examined for PI(4,5)P_2_ by deconvolution confocal microscopy. The phospholipid was detected by staining with an anti-PI(4,5)P_2_ primary antibody and visualized by indirect immunofluorescence using a TRITC-labeled secondary antibody (*red*) targeted to the primary antibody. Insets in **(C,D)** show Gag expression in the cells as detected using primary antibody that recognized the capsid domain in Gag and a FITC-labeled secondary antibody targeted to the primary. The bar measures 10 μm. **(E)** Distribution of PI(4,5)P2 in cells.

Store Ca^2+^ release is essential for a large number of cellular processes (Clapham, 2007). Both the continuous requirement for IP3R mediation of store Ca^2+^ release (cf. Figure 3) and the high level of cytosolic Ca^2+^ originating from internal stores (cf. Figure 2 and Ehrlich et al., 2011) would suggest that the process of Gag assembly empties the Ca^2+^ store. Thus, cells supporting productive Gag particle formation have either adapted to operating on empty stores or engage in store refilling. Store refilling is characterized by the presence of plasma membrane-proximal puncta that are recognized to consist of ER-resident STIM1 with its C-terminal tail engaged in interaction with PM-resident PI(4,5)P_2_ headgroups and the Orai Ca^2+^ influx channel (Walsh et al., 2009; Carrasco and Meyer, 2011). We therefore used plasma membrane PI(4,5)P_2_ as an indicator of store refilling to compare its status in naïve COS-1 cells and cells supporting productive and non-productive Gag VLP formation. Figure 9 shows the plasma membrane proximal (z plane = 0 μm) and interior (z plane = 1.2 μm) regions of cells examined by deconvolution confocal microscopy using an anti-PI(4,5)P_2_ antibody. PI(4,5)P_2_ was detected in the cell interior in all cases, i.e., whether the cell was mock-treated (panel **A**), expressed WT Gag constitutively (panel **B**) or transiently (panel **C**), or expressed P7L-Gag (panel **D**). However, the phospholipid was apparent in a plasma membrane-proximal location only in the cells expressing WT Gag (panels **B,C**). A quantitative analysis of 30 cells (panel **E**) indicated that PI(4,5)P_2_ was detected at or near the plasma membrane (*filled symbol*) in the cells expressing Gag at a ~3- to 5-fold higher frequency than in the mock-treated cells or the cells expressing the P7L-Gag mutant (*n* = 2). Taken together, the results suggest that, compared to mock-transfected cells or cells expressing P7L, more of the cells supporting productive Gag particle formation have the components for store-refilling in place. As this condition requires a functional PTAP L domain, these observations implicate Tsg101 binding in the store refilling event related to Gag assembly.

**Figure 10:**
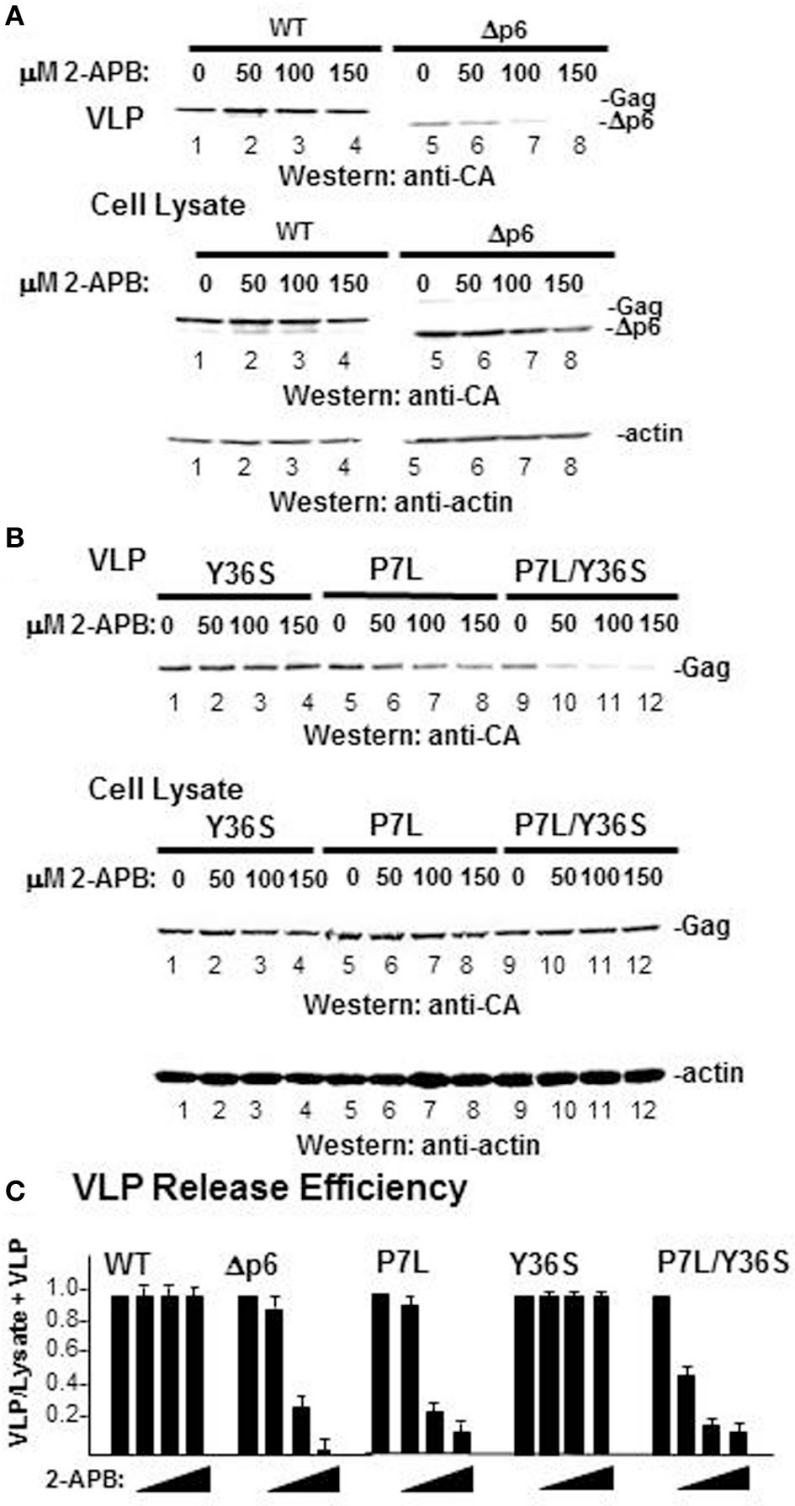
**L domain-dependent resistance to 2-APB-mediated inhibition of SOCE**. COS-1 cells were transfected with DNA encoding: **(A)** WT Gag (WT, *lanes 1–4*) or a Gag mutant missing the entire C-terminal p6 region (Δ p6, *lanes 5–8*); **(B)** Gag with a Tyr36 to Ser substitution in L Domain-2 in p6 (Y36S, *lanes 1–4*); Gag with Pro7 to Leu substitution in L Domain-1 (P7L, *lanes 5–8*); or Gag with both substitutions (P7L/Y36S, *lanes 9–12*). At 24 h post-transfection, the tissue culture media was replaced with treatment media containing DMSO or increasing concentrations of 2-APB. After 24 h, tissue culture media and cells were harvested for Western analysis of Gag VLP (*top*), cell-associated Gag (*middle*), and actin (*bottom*). **(C)** VLP release efficiency. To facilitate sample comparison, more cells were used for samples containing the P7L and P7L/Y36S mutants.

To further examine the relationship between viral budding and store refilling, the effect of blocking store refilling on VLP production was tested (Figure 10). All cells undergo store refilling with the store operated Ca^2+^ entry (SOCE) as the ubiquitous refilling mechanism that relies on functional and physical coupling between the ER-resident Ca^2+^ sensor protein (STIM1) and the plasma membrane-resident Orai Ca^2+^ influx channel (Smyth et al., 2010; Vaca, 2010). The agent, 2-APB at 50 μM and higher concentrations potently and selectively inhibits Ca^2+^ influx by interrupting this coupling (Cheng et al., 2011; Yamashita et al., 2011). As shown in panel **(A)**, WT Gag VLP release was not perturbed by 2-APB treatment (*lanes 1–4*). Following deletion of the entire L domain-bearing p6 region (Δp6-Gag), Gag exhibited dose-dependent 2-APB sensitivity (*lanes 5–8*). To map the determinant of resistance to 2-APB within the p6 region, a test of the effect of the treatment was done using Gag L domain mutants (panel **B**). The p6 region bears both L domain-1 (PTAP), which recruits Tsg101 (Garrus et al., 2001; Martin-Serrano et al., 2001; VerPlank et al., 2001) and L-domain-2 (LY_36_PX_*n*_L), which recruits Alix (Martin-Serrano et al., 2003; Strack et al., 2003; von Schwedler et al., 2003). To determine the 2-APB sensitivity of budding directed mainly by L domain-1, L domain-2 was impaired with a Ser substitution for Y36. This single mutation in Gag was sufficient to abrogate Alix binding (Watanabe et al., 2013). The Y36S mutation had little effect on budding efficiency in the presence or absence of 2-APB (*lanes 1–4*). In contrast and as previously reported (Ehrlich et al., 2011), the P7L mutant exhibited dose-dependent sensitivity to 2-APB (*lanes 5–8*). Although the Y36S mutation in itself had little impact on release efficiency, when combined with the P7L mutation the resulting Gag double mutant (P7L/Y36S) is released at very low efficiency (Medina et al., 2011). Under this condition, budding is directed by determinants in the C-terminal region of the CA domain in Gag and facilitated by the ubiquitin E3 ligase Nedd4-2s (Chung et al., 2008; Usami et al., 2008). Interestingly, the double mutant P7L/Y36S exhibited greater sensitivity to 2-APB than either parent alone (*lanes 9–12*). Thus, addition of 2-APB reduced VLP release efficiency for all budding pathways except the one mediated by Tsg101 (panel **C**). That WT Gag VLP production was able to circumvent the block to STIM1-Orai coupling imposed by 2-APB reflects functional modulation of the store refilling mechanism by the Gag-Tsg101 complex.

## Discussion

The N-terminal MA domain in the Gag polyprotein is the major determinant of plasma membrane binding (reviewed in Chukkapalli and Ono, 2011). The positive charges in MA, the N-terminal myristate moiety and the PI(4,5)P_2_ binding pocket in MA all contribute significantly to Gag targeting and plasma membrane binding. Based on previous studies *in vitro* using model membranes, MA can be expected to preferentially direct Gag binding to acidic phospholipids (Ehrlich et al., 1996; reviewed in Scarlata and Carter, 2003; Chukkapalli and Ono, 2011). Given that the main components of rafts are uncharged, we previously suggested that MA would direct Gag outside of rafts (Scarlata and Carter, 2003). A recent report documents the exclusion of multimerized Gag from ordered membrane domains, i.e., lipid rafts, in model membranes (Keller et al., 2013). The authors suggested that stable plasma membrane localization of Gag might be more complex than previously thought and possibly involve additional protein machinery. Supporting this notion, there is increasing evidence that the raft-enriched membrane domain from which Gag assemblages are known to bud is derived through Gag-initiated plasma membrane alterations (Krementsov et al., 2010; Ono, 2010; Kerviel et al., 2013). If so, then the targeting of Gag to PI(4,5)P_2_, a phospholipid found in both raft and non-raft domains (Calloway et al., 2011), may only be one of several events required to secure stable localization of Gag at the plasma membrane. Our studies demonstrate that IP3R-mediated Ca^2+^ signaling is required for stable plasma membrane localization of Gag (Ehrlich et al., 2010, 2011). This makes IP3R a component of the additional protein machinery alluded to by Keller et al. (2013).

As noted above, we initially identified IP3R and other Ca^2+^ signaling proteins through a proteomic search for proteins associated with Gag, but not a L-domain mutant, in a membrane-enriched subcellular fraction. The goal of that study was identification of cellular factors that participate with HIV-1 Gag and ESCRT in facilitating the budding event. Here, we show functional linkages between IP3R and Tsg101, the ESCRT-1 component recruited by the PTAP L domain motif in Gag. Compared to control cells, cells expressing Gag exhibited a higher level of cytosolic Ca^2+^ originating from the ER Ca^2+^ store, suggesting that Gag induced the change. This Gag-associated activity required an intact PTAP motif (cf. Figure 2), corroborating an earlier finding based on measurement of cytosolic Ca^2+^ in single cells (Ehrlich et al., 2011). Consistent with the elevated Ca^2+^ level detected in Gag-expressing cells, stable Gag association with the plasma membrane was found to require continuous IP3R function (cf. Figure 3). Since in almost all cell types, the IP3R channel serves as the principal means of mobilizing store Ca^2+^ (cf. Figure 1; Hanson et al., 2004; Patterson et al., 2004a,b; Banerjee and Hasan, 2005; Clapham, 2007; Mikoshiba, 2007), more of the WT Gag-expressing cells were in store refilling mode compared to the P7L-Gag-expressing cells (cf. Figure 9). Moreover, Gag resistance to 2-APB, an inhibitor of store-refilling, exhibited PTAP L domain-dependence (cf. Figure 10). As reported previously, adventitiously-expressed IP3R-FLAG was detected at the periphery of cells expressing Gag, but only when the PTAP motif was intact (Ehrlich et al., 2011). A similar PTAP-dependent IP3R redistribution was also observed in the current study, as significantly more gold-tagged IP3R was detected at the periphery of cells transfected with Gag compared to mock-transfected cells (cf. Figure 5). Gold-tagged IP3R was also detected in association with VLPs (cf. Figure 5), consistent with a previous observation that IP3R is encapsidated in virions released from HIV-1-infected macrophages (Chertova et al., 2006). Interestingly, in that study, the virion-incorporated IP3R was recovered from a band in a SDS-PAG slice that exhibited a molecular mass of 100–130 kDa which is similar to the mass of the IP3R-related protein that was predominant in our study (cf. Figures 7, 8). As IP3R redistribution to the cell periphery occurs in response to specific stimulus rather than to bulk changes in ER structure (Vermassen et al., 2004; Chalmers et al., 2006), the results suggest that Gag is a stimulus for IP3R translocation to the cell periphery. Together, these findings show that modulation of ER Ca^2+^ release and ER store refilling are aspects of productive Gag assembly and that these events are facilitated by recruitment of Tsg101 by Gag.

Taken together, our findings show that stable plasma membrane localization of Gag requires release of store Ca^2+^. It is possible that some of the released Ca^2+^ maintain continuous IP3R activation itself since Ca^2+^ is an activator of PLC, the enzyme catalyzing the hydrolysis of PI(4,5)P_2_ to produce the ligand (IP3) needed to activate IP3R (Rhee, 2001; Suh et al., 2008). Ca^2+^ may also play a role in the competency of Gag to bind membranes. It is known that the lipid moiety, myristate, which is co-translationally added to the second N-terminal residue in MA, contributes to Gag membrane binding (reviewed in Chukkapalli and Ono, 2011). *In vitro* membrane binding studies show two conditions that favor solvent exposure of myristate: (i) protonation of His89 in the MA domain in Gag (Fledderman et al., 2010) and (ii) Ca^2+^-dependent binding of calmodulin to MA (Ghanam et al., 2010). IP3R-mediated Ca^2+^ store release can therefore be expected to promote the exposure of myristate, especially when it occurs at or near the plasma membrane.

Recent evidence indicates that the junction between the plasma membrane and adjacent ER allows for rapid spatially restricted Ca^2+^ signaling (reviewed in Carrasco and Meyer, 2011). We can detect Gag and ER in the TIRF field (cf. Figure 4); immuno-gold-labeled IP3R in VLPs (cf. Figure 5); IP3R-Gag fluorescence co-localization (cf. Figure 6); reciprocal co-IP of Gag and an IP3R-related protein (cf. Figure 7); and Gag-IP3R floatation to the same light membrane fraction in sucrose gradients (cf. Figure 8) indicating that Gag and IP3R are in proximity at the cell periphery, i.e., at ER-PM junctions. Keeping an appropriate Ca^2+^ level in this restricted space near Gag assembly sites appears to be necessary to maintain Gag on the PM (cf. Figure 3). Possibly, an as yet unidentified Tsg101 binding partner that is also capable of associating with IP3R links IP3R to the assembling Gag-Tsg101 complexes in this region. For example, among proteins capable of associating with the receptor, junctophilin proteins, which are essential components of ER-PM junctions, conserve a Tsg101 recognition sequence (PTXP) (Stamboulian et al., 2005). Also, members of the Src family of non-receptor tyrosine kinases have been shown to bind both IP3R and Tsg101 (Jayaraman et al., 1996; Tu et al., 2010). Early studies showed that IP3R channel activity is increased by addition of Src family kinases *in vitro* (Jayaraman et al., 1996). Tsg101 has been shown to be responsible for Src delivery to the cell periphery (Tu et al., 2010). PLC gamma also binds activated Src (Zachos et al., 2013). It has been reported that, if Src phosphorylates IP3R at Tyr353 located in the IP3R binding core, the affinity for IP3 is increased (Cui et al., 2004). The phosphorylated IP3R is more sensitive to IP3 and less sensitive to Ca^2+^-mediated inhibition (deSouza et al., 2007). The combined effect permits the channel to be more open under a wide range of IP3 and Ca^2+^ concentrations. Thus, proteins like junctophilin or Src family members could function as tethers that permit Tsg101 to modulate IP3R gating.

In summary, we propose a model wherein Gag-recruited Tsg101 serves as agonist for redistribution of IP3R to the cell periphery. Within the confines of the ER-PM junction, close proximity between the Gag-Tsg101 complex and IP3R channels allow physical associations that alter opening, release of store Ca^2+^, and store-refilling. The resulting elevation of Ca^2+^ level in the immediate vicinity of the plasma membrane drives events that lead to stable membrane localization of assembling Gag. In this model, Tsg101 has a novel role that relates to modulation of the gating of the ER store and store refilling. Our findings support a paradigm for HIV-1 assembly wherein both intact and hydrolyzed PI(4,5)P_2_ are required. Identification of Ca^2+^ signaling machinery as a component of the PTAP L domain function in viral budding may provide new targets for development of anti-viral strategies aimed at this aspect of virus replication.

### Conflict of interest statement

The authors declare that the research was conducted in the absence of any commercial or financial relationships that could be construed as a potential conflict of interest.
